# A Promising Method of Typhoon Wave Retrieval from Gaofen-3 Synthetic Aperture Radar Image in VV-Polarization

**DOI:** 10.3390/s18072064

**Published:** 2018-06-28

**Authors:** Qiyan Ji, Weizeng Shao, Yexin Sheng, Xinzhe Yuan, Jian Sun, Wei Zhou, Juncheng Zuo

**Affiliations:** 1 Marine Science and Technology College, Zhejiang Ocean University, Zhoushan 316022, China; jiqiyan@zjou.edu.cn (Q.J.); shengyexin@hotmail.com (Y.S.); zjuncheng@zjou.edu.cn (J.Z.); 2 National Satellite Ocean Application Service, State Oceanic Administration, Beijing 100081, China; harley_yuan@mail.nsoas.org.cn; 3 Physical Oceanography Laboratory/CIMST, Ocean University of China and Qingdao National Laboratory for Marine Science and Technology, Qingdao 266100, China; sunjian77@ouc.edu.cn; 4 South China Sea Institute of Oceanology, Chinese Academy of Sciences, Guangzhou 510301, China; zhouwei@scsio.ac.cn

**Keywords:** typhoon wave, VV-polarization, Gaofen-3 synthetic aperture radar

## Abstract

The motivation of this work is to explore the possibility of typhoon wave retrieval (the main parameter is significant wave height (SWH)) for C-band Gaofen (GF-3) synthetic aperture radar (SAR) with a wide swath coverage (>400 km). We aim to establish an analysis of a typhoon wave in the subresolution-scale (approximately 20 × 20 km^2^) on GF-3 SAR through SAR-measured parameters, including a normalized radar cross section (NRCS) and variance of the normalized SAR image (herein called cvar), which are the basic variables in an empirical wave retrieval algorithm and are independent of visible wave streaks. Several typhoons around the China Seas were captured by Chinese GF-3 SAR in 2017; e.g., Noru, Doksuri, Talim and Hato. The wave fields simulated from the third-generation numerical wave model WAVEWATCH-III (WW3) are collocated with these images. In general, the distribution patterns of the typhoon waves from the WW3 model are consistent with wave fields from the European Centre for Medium-Range Weather Forecasts (ECMWF) at 0.125° grids, indicating that the simulation results from the WW3 model are suitable for our study. In addition to winds retrieved from GF-3 SAR images in vertical-horizontal (VH) polarization, the characteristics of the typhoon wave on vertical-vertical (VV) polarization GF-3 SAR images are studied. It is found that SWH has a linear relationship with NRCS and cvar, however, SWH fluctuates with wind speed at all incidence angles. Based on the analyzed results, we simply tune two empirical wave retrieval algorithms for GF-3 SAR in typhoons. Although the correlation (COR) reaches 0.5 taking account into the NRCS term, a more accurate retrieval algorithm, including more related terms, is anticipated for further development for GF-3 SAR and validated through more typhoon images.

## 1. Introduction

Typhoons have a significant influence on the heat, water vapor, and material exchange between the ocean and the atmosphere, and they are considered to be an important dynamic phenomenon of oceanographic study. Due to the risks involved in measuring waves during a typhoon, it is rather difficult to use traditional technology; e.g., moored buoy and sailing ship. Space-borne synthetic aperture radar (SAR) can obtain high spatial resolution information of the sea surface with a large swath coverage following its orbit. Therefore, currently SAR is an important, direct, and effective technical way to obtain marine data [1]. Moreover, recent research has shown that SAR has the capability of wind [2,3] and wave [4] monitoring in typhoons.

To date, theoretically-based wave retrieval algorithms have included the “Max-Planck Institute” algorithm (MPI) [5,6,7], the Semi Parametric Retrieval Algorithm scheme (SPRA) [8], the Partition Rescaling and Shift Algorithm (PARSA) [9], and the Parameterized First-guess Spectrum Method (PFSM) [10,11,12]. These algorithms are based on the wave mapping mechanism on SAR; e.g., tilt modulation, hydrodynamic modulation [13] and velocity bunching [5]—which require a first-guess wave spectrum as prior information because velocity bunching is a non-linear modulation causing signal loss in the flight direction. The MPI and PARSA algorithms rely on the simulation from a numerical ocean wave model, while SPRA and PFSM perform the computation by using parametric functions such as the Jonswap function [14]. The PFSM algorithm is more practically applicable than other algorithms, because it employs a SAR-derived wind speed to produce the first-guess wave spectrum. Moreover, the non-linear wind-sea and the linear-mapping swell spectrum are first separated in the scheme of the PFSM algorithm, indicating that wind-sea and swell can be inverted through corresponding portions by using different methods. Also, empirical algorithms have been developed aimed at wave retrieval from SAR at C-band, such as CWAVE_ERS [15], CWAVE_ENVI [16], CSAR_WAVE [17], and QPCWAVE_GF3 [18]. The advantage of these algorithms is that wave parameters can be directly inverted from SAR images without calculating the complex modulation transfer function (MTF) of each mapping modulation. However, these algorithms were developed and validated at low and moderate sea states [19].

Wind is an essential parameter in typhoons. The geophysical model function (GMF) is widely used for wind retrieval from SAR. GMF at C-band includes CMOD4 [20], CMOD-IFR2 [21], CMOD5 [22] with CMOD5N for neutral wind [23], and C-SARMOD [24]. It is reported in [25,26,27,28,29,30] that the root mean square error (RMSE) of SAR-derived wind speed is within 2 m/s for various C-band SAR at low and moderate winds. GMF has a worse performance in strong winds (probably greater than 25 m/s) because the backscattering signal in the co-polarization channel (vertical-vertical (VV) and horizontal-horizontal (HH)) encounters saturation problems under such wind conditions [31,32]. This makes wave retrieval difficult as strong winds are a significant aspect of typhoons. Furthermore, the non-linearity of velocity bunching is higher in typhoons than that at low and moderate sea states. Thus, a theoretically-based wave retrieval algorithm is not applicable because a SAR spectrum is useless with a cutoff of waves. When developing a wave retrieval algorithm in typhoons, it is essential to analyze in advance the characteristic effects of typhoon waves on SAR data.

The Chinese Gaofen-3 satellite carrying a SAR sensor at C-band was launched by the China Academy of Space Technology (CAST) in August 2016. During a mission in 2017, data from several typhoons were captured and recorded in the China Sea; e.g., Noru, Doksuri, Talim and Hato. These images were acquired in dual-polarization (VV and VH) and officially delivered by the National Satellite Ocean Application Service (NSOAS). It was found that the SAR backscattering signal encountered a saturation problem under strong wind conditions (probably greater than 25 m/s) [32], while the wind speed saturation at the VH-polarization channel could be as high as 55 m/s [33,34], indicating that strong winds can be effectively retrieved in VH-polarization. Recently, several wind retrieval algorithms for VH-polarization SAR have been developed [35,36,37,38,39,40,41] through the C-band Radarsat-2 and Sentinel-1 images with collocated referred winds. In our previous study [42], a technique for typhoon wind retrieval from VH-polarization GF-3 SAR image was also proposed. The RMSE of wind is around 5 m/s when retrieved winds are compared with the measurements from Windsat at 0.25° grid and simulations from the Global and Regional Assimilation and Prediction System—Typhoons model (GRAPES-TYM) at 0.12° grid.

In our work, five VV-polarization GF-3 SAR images including apparent typhoon eyes (TE) have been compared with computations from the numerical wave model WAVEWATCH-III (WW3). Through the collocated dataset, the relationships between significant wave height (SWH) and several SAR-measured parameters were investigated, including strong winds retrieved from VH-polarization GF-3 SAR images, variance of the normalized SAR image (herein called cvar), and normalized radar cross section (NRCS). These are related with the sea state and can be obtained without visible wave streaks on the SAR images. As a preliminary study, we propose two empirical wave retrieval algorithms for GF-3 SAR in typhoons.

The remaining parts of this paper are organized as follows: Data collection are described in Section 2, including GF-3 SAR images and referred sources. The methodology for an ocean wind retrieval algorithm for GF-3 SAR is introduced and simulated wave fields from the WW3 model in typhoons are exhibited in Section 3. The analysis of typhoon waves from GF-3 SAR is described in Section 4. We provide the tuned wave retrieval algorithms in Section 5, and the conclusions are summarized in Section 6.

## 2. Dataset Collection

**Figure 1:**
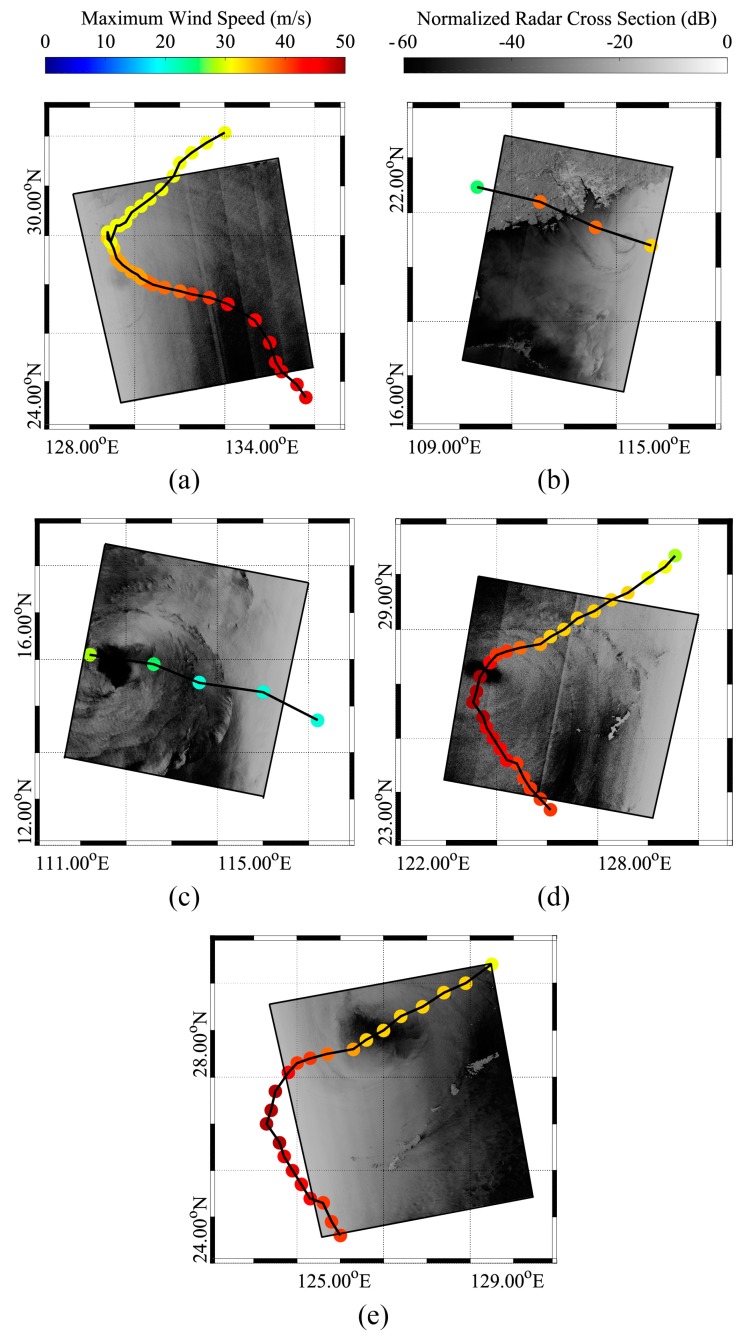
The quick-look image of VV-polarization C-band Gaofen (GF-3) synthetic aperture radar (SAR) images overlaid with the tracks of typhoons after calibration. (**a**) The image from typhoon Noru acquired in Global Observation (GLO) mode on 4 August 2017 at 09:12 UTC; (**b**) The image from typhoon Hato acquired in Wide ScanSAR (WSC) mode on 22 August 2017 at 22:23 UTC; (**c**) The image from typhoon Doksuri acquired in WSC mode on 13 September 2017 at 22:14 UTC; (**d**) The image from typhoon Talim acquired in GLO mode on 14 September 2017 at 21:29 UTC; (**e**) The image from typhoon Talim acquired in WSC mode on 16 September 2017 at 09:34 UTC.

In this study, five GF-3 SAR images acquired in Global Observation (GLO) and Wide ScanSAR (WSC) mode are used. These images were taken in dual-polarization (VV and VH) and processed to a Level-1B (L-1B) product during the period of typhoons Noru, Doksuri, Talim, and Hato in 2017, when the maximum wind speed reached 40 m/s. The quick-look images of the collected images in VV-polarization channel after calibration are presented in Figure 1, on which are overlaid the track of each typhoon provided by the Japan Meteorological Agency (JMA). Complex descriptions of these five images and information about the typhoons are listed in the Appendix A (Table A1). Equation (1) is used to calculate the NRCS of VV-polarization GF-3 SAR acquired in L-1B mode:

(1) $$\sigma_{\mathrm{VV}}^{0}{=\mathrm{DN}}^{2}{\left(\frac{M}{65535}\right)}^{2}-N\;[\mathrm{dB}]$$

where $\sigma_{\mathrm{VV}}^{0}$ is the NRCS in units of dB, *DN* is SAR-measured intensity, *M* is the external calibration factor and *N* is the offset constant stored in the annotation file. It should be noted that those images acquired in GLO and WSC mode comprise several strip-beams that are slightly lighter near their edges because of the effect of instrumental noise. Although there are a few time differences between the SAR imaging time and the track data, it is shown that GF-3 SAR has the ability to capture typhoons. 

We also show the five GF-3 SAR images in the VH-polarization channel corresponding to each image in Figure 1. Typhoon winds are retrieved using an empirical algorithm and were exhibited in Figure 8 in our recent study [42], showing an approximate 5.1 m/s RMSE of wind speed as validated against strong wind simulations from GRAPES-TYM. SAR-derived winds are taken as the initial information when making a data analysis in our work. 

In order to study the performance of typhoon waves on GF-3 SAR, we initially collected the wave data from European Centre for Medium-Range Weather Forecasts (ECMWF) reanalysis datasets. Specifically, ECMWF continuously provide global atmospheric-marine reanalysis fields for the worldwide investigator, which have a fine spatial resolution (up to 0.125 × 0.125°) at an interval of 6-h daily. These data are considered to be reliable sources, which are widely used for developing and validating the algorithms for SAR retrieval; e.g., wind [22,23] and wave [12,30]. Unfortunately, we found that there was 2–3 h between the ECMWF interval time and the GF-3 SAR imaging time. Therefore, ECMWF reanalysis data at an interval of 6-hours is not directly used here.

**Figure 2:**
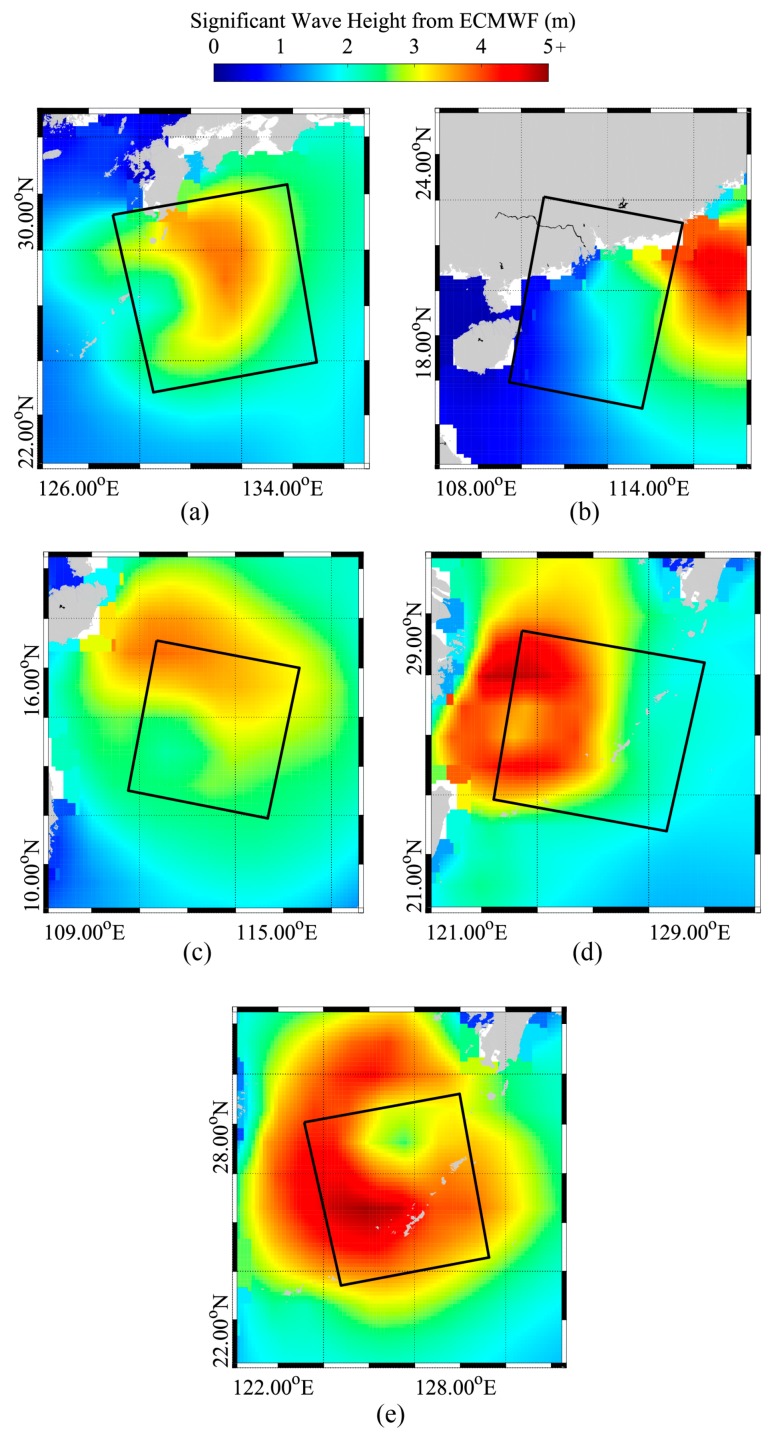
The significant wave height (SWH) maps image from European Centre for Medium-Range Weather Forecasts (ECMWF) reanalysis datasets overlaid with the spatial coverage of GF-3 SAR images. (**a**) The map on 4 August 2017 at 06:00 UTC for typhoon Noru acquired in GLO mode; (**b**) The map on 22 August 2017 at 18:00 UTC for typhoon Hato acquired in WSC mode; (**c**) The map on 13 September 2017 at 18:00 UTC for typhoon Doksuri acquired in WSC mode; (**d**) The map on 14 September 2017 at 18:00 UTC for typhoon Talim acquired in GLO mode; (**e**) The map on 16 September 2017 at 06:00 UTC for typhoon Talim acquired in WSC mode.

The third-generation numerical wave model, abbreviated as the WW3 model, was developed by the National Oceanic and Atmospheric Administration/National Centers for Environmental Prediction (NOAA/NCEP), which is in the spirit of the previous WAM model. Recent research has revealed the WW3 model to have a good performance when simulating the characteristics of typhoon waves [43,44]. Therefore, typhoon wave fields collocated with the five VV-polarization GF-3 SAR images are simulated using the WW3 model (the latest version 5.16) after taking 0.12° gridded winds from GRAPES-TYM [45] as forcing fields. The detailed model running configurations are presented in a later section. ECMWF reanalysis wave data at 0.125° grids are referred to as background information in order to qualitatively verify the applicability of the simulated waves from the WW3 model. Figure 2 shows the SWH maps of the ECMWF reanalysis data, which is nearest to the imaging time of each collected GF-3 SAR image in Figure 1. In addition, wave measurements from altimeter Jason-2 are used to quantitatively validate the simulated waves from the WW3 model.

## 3. Wind Retrieval Algorithm and Typhoon Waves Simulated by the WW3 Model 

As mentioned in the introduction, strong winds are an important contributor to typhoon waves. We briefly present the recent development for strong winds retrieval from VH-polarization GF-3 SAR images. In addition, typhoon waves at 0.1° grids, as simulated by the WW3 model, are shown.

### 3.1. Development of Strong Winds Retrieval from VH-Polarization GF-3 SAR Image

Through a collocated dataset, including three VH-polarization GF-3 images in GLO/WSC mode and wind simulations at 0.12° grids from GRAPES-TYM in typhoons, the dependence of wind speed and incidence angle on VH-polarization NRCS of GF-3 SAR was investigated, following which an empirical algorithm was developed in [42], which is stated as follows:

(2) $$\sigma_{\mathrm{VH}}^{0}=f_{1}\left(1+\alpha \|f_{2}\|\right)+\beta \;\left[\mathrm{dB}\right]$$

where

(3) $$f_{1}{=\mathrm{AU}}_{10}+B$$

*f*_2_ = C_1_*θ*^2^ + C_2_*θ* + C_3_ (4)

where $\sigma_{\mathrm{VH}}^{0}$ is the VH-polarization NRCS in units of dB, *f*_2_ is the function of radar incidence angle which is normalized into [−1, 1] and the coefficients α, β, A, B, and matrix C are the tuned constants.

The proposed algorithm has been implemented for several additional VH-polarization GF-3 SAR images. The inverted winds were compared with measurements from Windsat winds at 0.25° grids and GRAPES-TYM winds with wind speeds up to 40 m/s. It is reported in [42] that the RMSE of wind speed is around 5.5 m/s, indicating GF-3 SAR in cross-polarization is a promising potential technique for typhoon winds monitoring without encountering the signal saturation problem that exists in the traditional GMF methodology for co-polarization SAR [20,21,22,23,24]. It should be noted that this empirical algorithm was tuned and only validated for noisy VH-polarization GF-3 SAR images.

Five of those GF-3 SAR images were also taken in the VV-polarization channel with visible typhoon eyes (TEs). Therefore, the results proposed in [42] can be directly employed here and are used as prior information on winds in the analysis.

### 3.2. Typhoon Waves from WW3 Model 

In this study, we employed the WW3 model to simulate wave fields in typhoons with fine resolution in spatial and temporal scale. 

The forcing fields are 0.12° gridded winds from GRAPES-TYM, which were used in our recent study for developing a strong winds retrieval algorithm (see Figures 2 and 10 in [42]). The data of topography is collected from the British Oceanographic Data Centre (BODC) of the General Bathymetric Chart of the Oceans (GEBCO) with a one arc-minute of spatial resolution. The simulated region is set as longitude from 105° E to 140° E and latitude from 10° N to 35° N, which includes the spatial coverage of five GF-3 SAR images. The spatial resolution of the output results is set as 0.1° grids at an interval of 30-min, indicating a time difference between outputs of the WW3 model and SAR images to within 15-min. 

**Figure 3:**
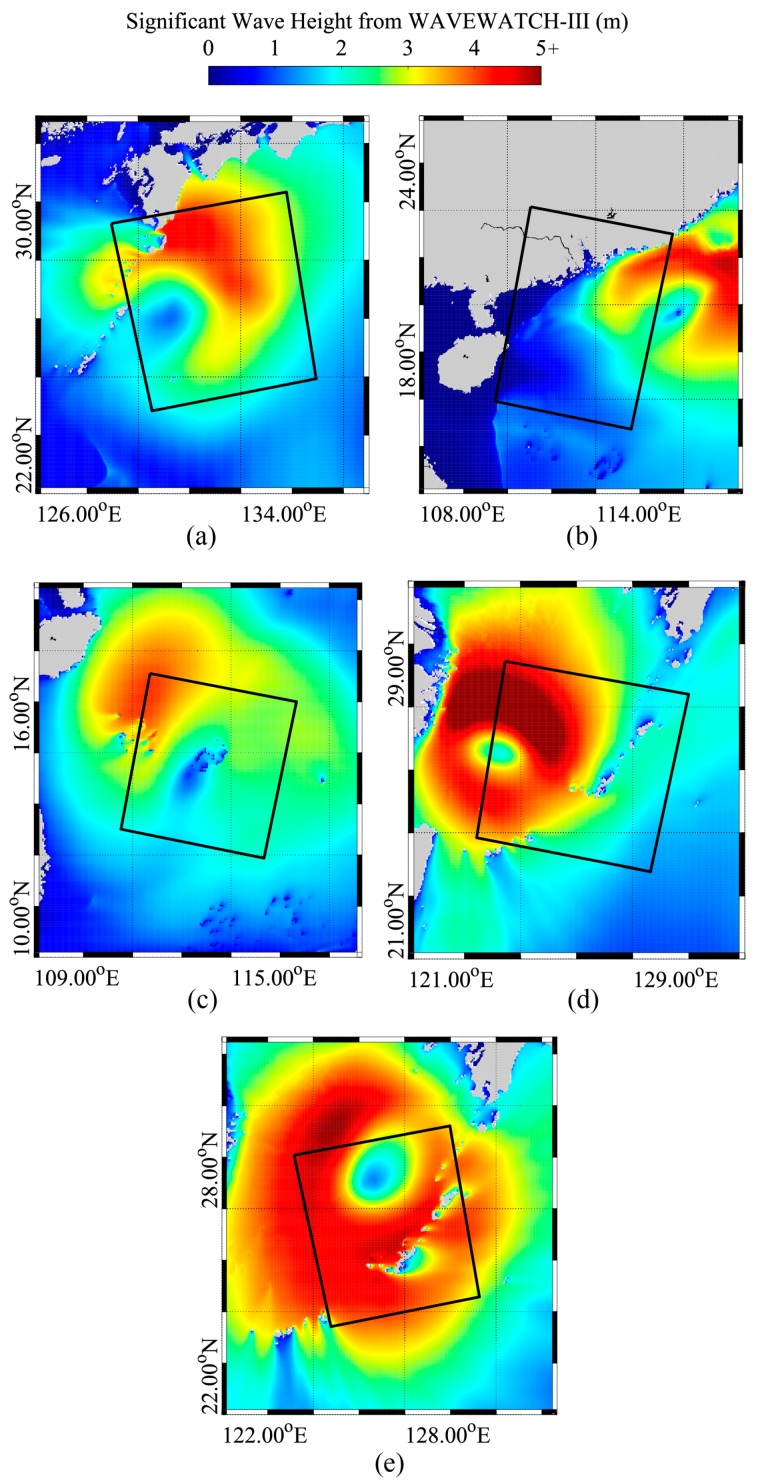
The simulated SWH maps of WAVEWATCH-III (WW3) model overlaid with the spatial coverage of GF-3 SAR images. (**a**) The map on 4 August 2017 at 09:00 UTC for typhoon Noru acquired in GLO mode; (**b**) The map on 22 August 2017 at 22:30 UTC for typhoon Hato acquired in WSC mode; (**c**) The map on 13 September 2017 at 22:00 UTC for typhoon Doksuri acquired in WSC mode; (**d**) The map on 14 September 2017 at 21:30 UTC for typhoon Talim acquired in GLO mode; (**e**) The map on 16 September 2017 at 09:30 UTC for typhoon Talim acquired in WSC mode.

Figure 3 shows the SWH maps simulated from the WW3 model overlaid with the spatial coverage of GF-3 SAR images, which is nearest the imaging time of five images. It should be noted that the time difference between simulations of the WW3 model and SAR imaging time is within 15 min. Obviously, the distribution patterns of simulated wave fields are consistent with ECMWF reanalysis data as exhibited in Figure 2. Moreover, the low sea states around the TEs are visible. Because of the higher spatial resolution setting for the WW3 model, the details of the wave fields are clearly observed, especially the waves across islands and coastal waters, indicating that simulated wave fields have been used appropriately here.

**Figure 4:**
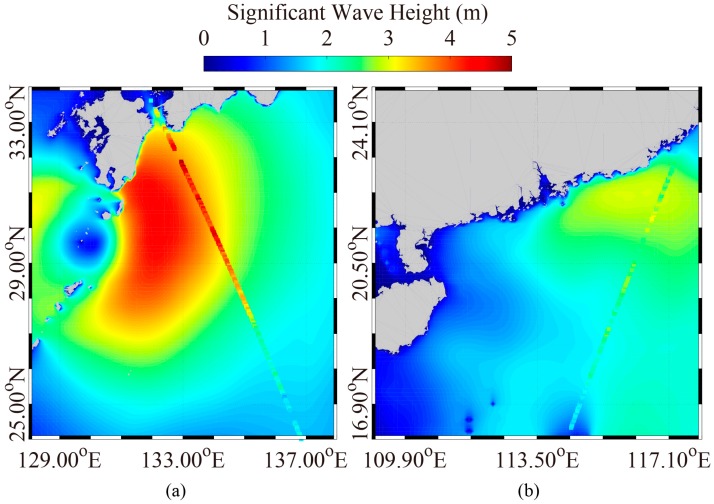
The simulated SWH maps of WW3 model overlaid with the footprint of altimeter Jason-2. (**a**) The map on 5 August 2017 at 06:00 UTC; (**b**) The map on 23 August 2017 at 17:00 UTC.

**Figure 5:**
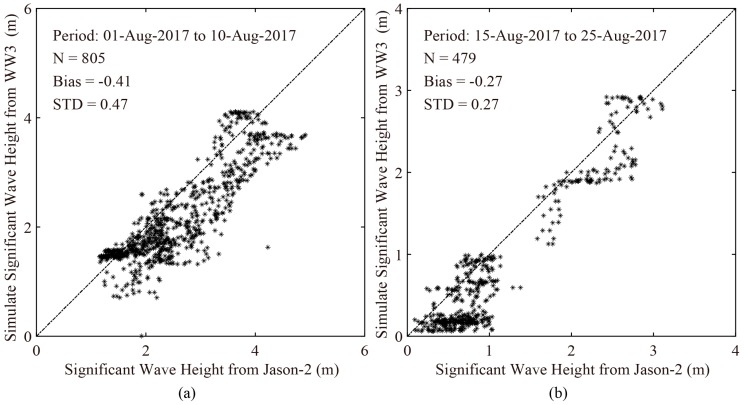
Comparisons between simulated results of WW3 model and available measurements from altimeter Jason-2 (**a**) over the duration of typhoon Noru; (**b**) over the duration of typhoon Hato.

We tried to collect the wave measurements from altimeter Jason-2 over the duration of the four typhoons; e.g., Noru, Doksuri, Talim, and Hato. Unfortunately, only the data of altimeter Jason-2 for typhoons Noru and Hato were available. As examples, the simulated SWH maps from the WW3 model taken on 5 August 2017 at 06:00 UTC and on 23 August 2017 at 17:00 UTC are shown in Figure 4, in which the colored rectangles represent the footprints of satellite altimeter Jason-2. The validation of SWH for the two typhoons is shown in Figure 5. The standard deviation (STD) of SWH is less than 0.5 m, indicating the simulated SWH from the WW3 model is suitable for this study.

## 4. Characteristics of Typhoon Waves on GF-3 SAR 

The sea state SWHs are supposedly related with winds at 10 m above the sea surface, NRCS united in dB and dimensionless cvar at a fixed incidence angle *θ*, which are assumed to be the basic variables in the empirical wave retrieval algorithms [4,15,16,17]. These parameters can be directly derived from a SAR intensity image and are independent of visible wave streaks, which do not regularly exist in typhoons. In this section, we analyze the relationships between SWH and wind, and NRCS and cvar in order to help in the development of a wave retrieval algorithm.

### 4.1. SAR Image Processing

In order to obtain a reasonable dataset, the whole VV-polarization GF-3 SAR image is divided into a number of square sub-scenes, with a spatial coverage of about 20 × 20 km^2^. Note that inhomogeneous sub-scenes due to heavy rainfall, where the ratio of image variance and squared image mean the value is greater than 1.05, are excluded [16,42,46]. Those sub-scenes covering the gridded data of the WW3 model at 0.1° are chosen. In total, more than 1500 matchups are available for this study. The dataset includes SWHs simulated by the WW3 model, the retrieved wind speed, NRCS, and cvar from five VV-polarization GF-3 SAR images.

### 4.2. Results

Figure 4a shows the relationship between simulated SWHs from the WW3 model and SAR-measured wind speeds. The colored lines represent the general tendency at the incidence angle to range from 10° to 50° for a 10° bin. It is recognized in [47] that the relative contribution of the wavelength range below 100 m to the SWH is of the order of less than 30% at winds greater than 20 m/s, indicating waves with longer wavelengths, including swell, are the dominant portion in a typhoon wave spectrum. This is the probable explanation for SWH fluctuation over wind speeds greater than 20 m/s.

**Figure 6:**
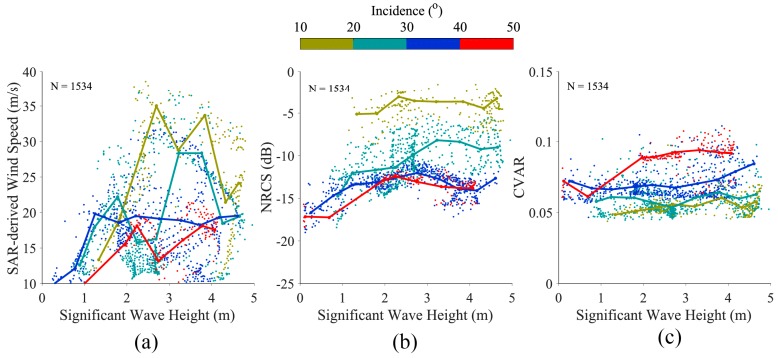
The relationship between simulated SWH from the WW3 model and three SAR-measured parameters, in which the colored lines represent the trend for each parameter at the incidence angle ranges from 10° to 50° for a 10° bin. (**a**) wind speed; (**b**) normalized radar cross section (NRCS); (**c**) cvar.

Interestingly, SWH has a linear relationship with NRCS and cvar, as shown in Figure 6b,c. This kind of behavior is also observed in [4] through several Radarsat-1/Envisat SAR images in HH-polarization and SWH simulations from numeric wave models. Moreover, it is found that NRCS has a negative relationship with incidence angles and cvar has a positive relationship with incidence angles at a fixed SWH. Although the SWH only reaches 5 m in our available dataset, it is acceptable that NRCS and cvar are directly related with SWH in typhoons. It is necessary to establish that the tendency is not obvious at low incidence angles smaller than 20°. This is reasonable because the Bragg backscattering mechanism on SAR is weak under such conditions.

## 5. Discussions

It appears that wave streaks are mostly invisible in a low spatial SAR image; e.g., more than 100 m for GF-3 SAR acquired in GLO and WSC mode—making it impossible to invert SAR intensity into an ocean wave spectrum under typhoon conditions. Considering the possible relationships mentioned above, a convenient approach for typhoon wave retrieval should be attainable, similar to the traditional GMF methodology for wind retrieval.

We simply propose two empirical wave retrieval algorithms for GF-3 SAR, taking a unqiue formulation as follows,

(5) $$\mathrm{SWH}=a\left(\begin{array}{c} \sigma_{\mathrm{VV}}^{0}\\ \mathrm{cvar} \end{array}\right)+b$$

where $\sigma_{\mathrm{VV}}^{0}$ is the NRCS in units of dB and coefficients *a* and *b* are the tuned constants, as shown in Table 1 for various radar incidence angle ranges, ranging from 10° to 50° for a 10° bin.

**Figure 7:**
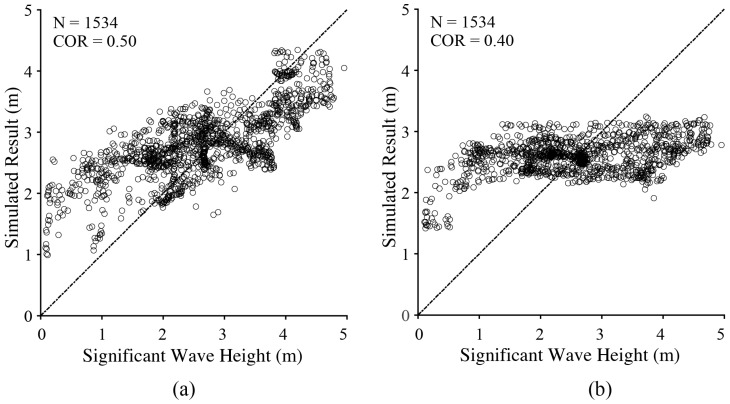
Simulated results of the entire dataset versus SWH from the WW3 model. (**a**) using an empirical algorithm including the NRCS term; (**b**) using an empirical algorithm including the cvar term.

Figure 7 shows the simulated results of the entire dataset versus SWH from the WW3 model. The correlation (COR) is 0.5 and 0.4 using an empirical wave retrieval algorithm including the NRCS term and cvar term, respectively. It is found that the performance of an algorithm including the NRCS term is better than that of an algorithm including the cvar term. In particular, the slope seems to be saturated when SWH is greater than 3 m using the algorithm that includes the cvar term. This is probably caused by a relatively small slope at the incidence angle of smaller than 30°, as exhibited in Figure 6c. Therefore, our study indicates that a more accurate empirical function for SWH retrieval in typhoons can be further developed similar to that in [4], in which the NRCS is assumed to be the main variable, together with other parameters; e.g., wind speed, cvar and incidence angle.

## 6. Conclusions

A cutoff wavelength in the azimuth direction is equivalent to the effect of non-linear velocity bunching on SAR. Theoretically, a cutoff wavelength is positively related with sea state SWH [5,6,17]. Therefore, non-linearity of velocity bunching in typhoons is higher than that at low and moderate sea states. In other words, long waves are cut off because the azimuthal cutoff wavelength is longer in typhoon conditions. On the other hand, a SAR image has a large swath coverage and somewhat reduced spatial resolution resulting in short waves being undetectable, which is usually more useful for typhoon monitoring; e.g., a 100–500 m pixel size for GF-3 SAR acquired in GLO and WSC mode. Collectively, because of poor quality of the SAR intensity spectrum in typhoons, typhoon wave retrieval is a challenge at present. Analyzing the characteristics of waves at subresolution-scale on SAR is essential work needed in the development of wave retrieval algorithm in typhoons.

Five GF-3 SAR images with apparent TEs during four typhoons are used in this study. The WW3 model is employed to simulate wave fields in those typhoons. The validation against measurements from altimeter Jason-2 shows a less than 0.5 m STD of SWH. A number of sub-scenes with a 20 × 20 km^2^ spatial coverage extracted from those images are collocated with SWH from the WW3 model at a 0.1° grid. Through the dataset, we study the relationship between SWH and three SAR-derived parameters at incidence angles from 10° to 50° for a 10° bin; e.g., wind speed, NRCS and cvar. Interestingly, it is found that SWH is linearly related with NRCS and cvar, which agrees with the conclusions proposed in [4]. Therefore, we simply tune two empirical algorithms for typhoon wave retrieval, taking a unique function. The two algorithms do not rely on a SAR spectrum and can be applicable without any prior information. COR is 0.5 between simulated SWH using the algorithm including the NRCS term and SWH from the WW3 model, while COR is 0.4 for the algorithm including the cvar term. Under this circumstance, we think this work gives an insight into the possibility of typhoon wave retrieval from GF-3 SAR image acquired in GLO and WSC mode, which has a swath coverage more than 400 km.

It is concluded that taking advantage of SAR-measured NRCS is a promising solution in the development of a typhoon wave retrieval algorithm. In fact, the case study in [48] for wave retrieval from an X-band SAR image taken in hurricane Sandy has already proved potentially effective after replacing NRCS derived winds in the algorithm XWAVE [47,49]. In future work, a more accurate algorithm can be further developed and then validated through more dual-polarization GF-3 SAR typhoon images, especially at extreme sea states.

## Figures and Tables

**Table 1:** Coefficients in (5), which are determined from the collocated data in our study.

	*θ*	10°~20°	20°~30°	30°~40°	40°~50°
NRCS Term	*a*	0.021	0.201	0.185	0.147
	*b*	3.531	4.769	5.000	4.991
cvar Term	*a*	43.557	5.197	17.623	29.397
	*b*	1.070	2.360	1.280	0.330

## Appendix A

**Table A1:** Information of five VH-polarization GF-3 SAR images and corresponding typhoons.

Typhoon	Acquisition Time (YYYY-MM-DD)	Incidence Range (°)	Pixel Size Azimuth × Range (m)	Swath Coverage (km)	Typhoon Eye (° E, ° N)
Noru	2017-08-04 09:11	13.15~45.76	72 × 400	650	131.1, 28.8
Hato	2017-08-22 22:22	14.26~41.17	18 × 80	500	114.4, 21.6
Doksuri	2017-09-13 22:13	20.56~43.75	18 × 80	480	112.2, 16.1
Talim	2017-09-14 21:29	14.20~45.76	72 × 400	650	124.8, 28.1
	2017-09-16 09:31	14.19~41.15	18 × 80	500	127.4, 29.3
